# Effect of Arbuscular Mycorrhizal Fungi, Selenium and Biochar on Photosynthetic Pigments and Antioxidant Enzyme Activity Under Arsenic Stress in Mung Bean (*Vigna radiata*)

**DOI:** 10.3389/fphys.2019.00193

**Published:** 2019-03-12

**Authors:** Mohammad Zahangeer Alam, Rebecca McGee, Md. Anamul Hoque, Golam Jalal Ahammed, Lynne Carpenter-Boggs

**Affiliations:** ^1^Department of Environmental Science, Faculty of Agriculture, Bangabandhu Sheikh Mujibur Rahman Agricultural University, Gazipur, Bangladesh; ^2^Department of Crop and Soil Sciences, Washington State University, Pullman, WA, United States; ^3^Department of Soil Science, Bangladesh Agricultural University, Mymensingh, Bangladesh; ^4^Grain Legume Genetics Physiology Research, Agricultural Research Service, United States Department of Agriculture, Pullman, WA, United States; ^5^College of Forestry, Henan University of Science and Technology, Luoyang, China

**Keywords:** AMF, Se, antioxidant, mung bean, arsenic

## Abstract

Environmental perturbations alter biochemical compounds in food crops. Arsenic (As), a toxic metalloid, is known to affect the cultivation of food crops in many regions of the world; however, the changes in chlorophyll, catalase (CAT), and proline in response to As stress and the role of stress relief substances remain largely unknown in mung bean (*Vigna radiate* L.). In this study, biochar (BC), arbuscular mycorrhizal fungi (AMF), and selenium (Se) were applied to soils as stress relief substances (under 30 mg kg^-1^ As stress), and the effects of BC, AMF, and Se on chlorophyll a, chlorophyll b, total chlorophyll, CAT activity, and proline content were studied in different mung bean genotypes. Under As stress, the chlorophyll a, chlorophyll b, and total chlorophyll contents in BARI mung 3, BARI mung 5, and BARI mung 8 were found statistically similar. Meanwhile, CAT activity increased in comparison to the control due to the application of BC, AMF, and Se in mung bean crops. However, proline was found significantly lower in AMF, BC, and Se-treated mung bean. This indicates that oxidative stress was potentially minimized in As-stressed mung bean crops due to the application of these stress relief substances. Notably, AMF was relatively effective against As stress in comparison to BC and Se. It is concluded that BC, AMF, and Se are all highly effective in enhancing antioxidant defenses as well as the nutritional quality of mung bean crops under As stress.

## Introduction

Biochemical compounds in food crops fluctuate with the changes in plant physiological status. Significant changes of biochemical compounds are indicators of environmental perturbations that often cause the deterioration of both yield and quality of food crops (Tripathi and Gautam, 2007). Chlorophylls are key photosynthetic pigments that participate in energy production through photosynthesis for the growth and development of plants. A positive correlation between leaf chlorophyll content and photosynthesis often leads to higher yield of crops. Nonetheless, chlorophyll content is also an important stress index as a significant reduction in leaf chlorophyll content is a common phenomenon under stressful conditions (Dehshiri and Paknyat, 2014). Similarly, proline and catalase (CAT) both are sensitive indicators of salt and other environmental stresses in different food crops (Gharsallah et al., 2016). These biochemical parameters change during salt and drought as well as arsenic (As) stress conditions in food crops (Swarnakar, 2014; Sofo et al., 2015). Reactive oxygen species (ROS) are the by-products of aerobic metabolism, and these components are often confined to certain cellular compartments (Alscher et al., 1997; Apel and Hirt, 2004; Gill and Tuteja, 2010; Sharma et al., 2012). Stress conditions trigger ROS production, leading to the occurrence of oxidative stress in plant cells. The antioxidant enzymes are a group of important defense compounds that actively participate in the detoxification of ROS such as superoxide and H_2_O_2_, especially under stress conditions (Esfandiari et al., 2007; Jaleel et al., 2007). For instance, CAT is involved in scavenging H_2_O_2_ (Horemans et al., 2000) and thus contributes to the plant defense mechanism against oxidative stress (Gill and Tuteja, 2010; Cheynier et al., 2013).

Globally people’s health is at risk due to micronutrient malnutrition (Hunger Notes, 2016). Nutrition for human beings depends on the availability, accessibility, and utilization of quality foods (Keatinge et al., 2011; Popkin et al., 2012). Mung bean is a high-quality pulse crop with high nutritional value. This crop is able to fix nitrogen, which has a positive impact on soil fertility and productivity in subsequent crops (Graham and Vance, 2003). The production and consumption of more legumes could aid in the reduction of global warming, eutrophication, acidification, and land degradation (Davis et al., 2010). In addition, mung bean is an important source of protein, with twice the amount on average compared with cereal grains (Davis et al., 2010). This is why legume crops are considered as the major source of protein for resource-poor people around the world and have been referred to as ‘poor man’s meat.’ Therefore, enhancing the quality and utilization of legumes is one of the best ways to tackle protein/energy malnutrition and micronutrient deficiencies in developing countries. Mung beans provide different nutrients, including the following: manganese, potassium, magnesium, folate, copper, zinc, and multiple vitamins. Because of the high nutrient content, the mung bean is considered a useful defender against several chronic diseases such as anemia and age-related diseases, including heart disease, cancer, diabetes, and obesity. Mung bean is also considered a precious food among Indian communities due to its medicinal value since roughly 1,500 B.C. (Shanmugasundaram, 2002).

Currently, mung bean cultivation is affected by several environmental stresses, which cause enormous yield losses every year (Mickelbart et al., 2015). Arsenic contamination is one of the main abiotic stresses that limit plant growth and leads to the deterioration of food quality by its entry into the food chain. Over the last several decades, the severity of As stress has increased to become a global problem for the cultivation of various food crops (Bailey-Serres et al., 2012). Arsenic is a toxic and carcinogenic element that occurs widely in soil environments around the world (Alam et al., 2011). Soil contamination with As occurs through natural and anthropogenic pathways (Meharg and Rahman, 2003). Arsenic is known to have many toxic effects on humans and ranks first in the priority list of hazardous substances (Agency for Toxic Substances and Disease Registry [ATSDR], 2011). This metal has severe effects on seedling growth, root anatomy, lipid peroxidation, electrolyte leakage, H_2_O_2_ accumulation, root oxidizability, and the activities of antioxidant enzymes in mung beans. CAT activity decreases in response to As exposure and corresponds to the decrease of H_2_O_2_ content as well. Arsenic causes the reduction of root elongation in food crops due to the increase of lipid peroxidation (Singh et al., 2007). Arsenic-stressed plants show reduced growth and pigment content in food crops. Total chlorophyll, CAT, and ascorbic acid content are drastically reduced in food crops due to the imposition of excess metals or metalloids such as As, cadmium, and lead (Zengin and Munzuroglu, 2005; Srivastava and Sharma, 2013).

Most recently, biochar (BC) soil amendment has been widely reported for the reduction of As uptake and toxicity in plants. Under As stress, BC application increases plant growth, biomass, photosynthetic pigments, grain yield, and quality (Rizwan et al., 2016). Similarly, As-induced oxidative stress (generation of H_2_O_2_ and lipid peroxidation) in plants is reduced significantly by arbuscular mycorrhizal fungi (AMF) inoculation. The alleviation potential of AMF is more evident with the increase in severity of As stress. Colonization of AMF in food crops results in higher activity of the antioxidant enzymes [superoxide dismutase (SOD), CAT, and guaiacol peroxidase] and increases the concentrations of antioxidant molecules (carotenoids, proline, and α-tocopherol) (Sharma et al., 2017). In addition, selenium (Se) also has significant impact on the morphological and biochemical characteristics of food crops under As stress conditions. Application of Se increases growth and improves the chlorophyll, protein, and proline content in food crops (Pandey and Gupta, 2015).

Arsenic toxicity and its effects on biochemical parameters have been mostly evaluated in ferns and a few other plants (Singh et al., 2007). Some research has been conducted on the changes of biochemical parameters during the bioremediation process in mung bean crops under As stress conditions. In this study, we applied AMF, Se, and BC in As-contaminated soils for the evaluation of the changes of biochemical compounds in mung bean crops. It is assumed that bioremediation using AMF, Se, and BC is significantly important for increasing the growth and antioxidant defense mechanism in mung bean crops under As stress. Thus, it is hypothesized that different biochemical parameters as well as nutritional quality in food crops might be improved through the application of BC, AMF, and Se under As stress conditions.

## Materials and Methods

### Collection of Soil

Soil was collected from a farmer’s field in Bangladesh for the pot experiment. The soil of the study area is silty loam in the agro-ecological zone of the Old Meghna Estuarine floodplain of Bangladesh, which falls under the order of Inceptisols according to the USDA (United States Department of Agriculture) soil classification. The collected soil samples were brought into the research field of Environmental Science at Bangabandhu Sheikh Mujibur Rahman Agricultural University (BSMRAU). All soil samples were dried under sunlight and ground before being used in pots for growing mung bean crops.

### Procurement of Mung Bean Seeds

Six mung bean genotypes are developed by Bangladesh Agricultural Research Institute (BARI). Among these genotypes, BARI mung 3, BARI mung 4, BARI mung 5, BARI mung 6, BARI mung 7, and BARI mung 8 were procured from the Pulse Research Center of BARI. The average yields of these varieties are 1.2–1.7 metric tons per ha. Total duration required from seed to maturity is about 60 to 65 days. The production season of these mung bean genotypes is from April to September in Bangladesh. These varieties were chosen in this study based on their height, life cycle, growing season, and yield.

### Biochar (BC), Fertilizers, Brick Pots, and Pesticides

Raw materials for the preparation of BC such as cow dung, sawdust, and rice husk were bought from the local market in Bangladesh. Then, the rice husk, cow dung, and sawdust BC were manufactured using a BC stove. This BC is produced by thermal decomposition of biomass under oxygen-limited conditions (pyrolysis). The characteristics of BC are influenced mainly by temperature and biomass. Higher pyrolysis temperature often results in an increased surface area and carbonized fraction of BC, leading to a high sorption capability for pollutants (Tang et al., 2013; Mia et al., 2015). Based on the total biomass (soils) in the pots, 4% BC were used in each treated pot for growing mung bean crops. Different fertilizers such as urea, triple super phosphate (TSP), muriate of potash (MOP) and Tricho derma-enriched biofertilizers were collected as a source of nitrogen (N), phosphorous (P), potassium (K), and other micronutrients. In addition, pots made of brick, fungicides (rubral), and chlorpyrifos were bought from the local market in Bangladesh.

### Preparation of Samples for Chemical Analysis

The collected soil samples from the farmer’s field in Bangladesh were brought into the Department of Environmental Science at BSMRAU. Initial soil samples of 250–300 (g) were taken from each composite using the guidelines of the Bangladesh Agricultural Research Council [BARC] (2012). The soil was air-dried at room temperature in the laboratory. Samples were then ground and sieved with a ≤250 μm mesh and preserved in polythene bags with proper labeling. Trichoderma-enriched biofertilizers and different BC samples were also prepared for chemical analysis as well as the soil samples.

### Analysis of N, P, K, As, Organic Carbon (OC) in Biochar and Other Samples

Total N percentage of the soil, Trichoderma-enriched biofertilizer, and BCs were determined by the Kjeldahl method (Jackson, 1973). The percentages of available P of the soil, Trichoderma-enriched biofertilizer, and BCs were analyzed by the Olsen method (Olsen and Sommers, 1982). The percentages of exchangeable K of the soil, Trichoderma-enriched biofertilizer, and BCs were estimated by the ammonium acetate extraction method (Jackson, 1973). Organic carbon (OC) as a percentage in the soil, Trichoderma-enriched biofertilizers, and BCs were detected by the wet oxidation method (Walkley and Black, 1935). Cow dung, sawdust, rice husk BC, Trichoderma-enriched biofertilizer, and background soils were digested separately following the heating block digestion procedure for the determination of total As concentration in these samples (Rahman et al., 2007). The total As in soils, Trichoderma, and BCs samples was analyzed by flow injection hydride generation atomic absorption spectrophotometry (FI-HG-AAS, Perkin Elmer A Analyst 400) using external calibration (Welsch et al., 1990) (Table 1).

**Table 1:** Total arsenic (As), percentage of OC, N, P, and K in background soil, Trichoderma-enriched biofertilizer, different biochars and applied fertilizers.

Samples	Total arsenic (As) in mg/kg	% Organic carbon (OC)	% Nitrogen (N)	% Phosphorus (P)	% Potassium (K)
Cow dung bio-char	0.044	15.88	1.19	1.28	1.42
Trichoderma-enriched biofertilizer	0.04468	14.50	1.28	1.20	0.87
Background soil sample	0.0784	0.39	0.05	0.001	0.003
Sawdust biochar	0.00349	24.60	0.33	0	0.77
Rice husk biochar	0.02499	6.32	0.30	0.10	0.33
Urea	-	-	0.0115	-	-
Triple super phosphate (TSP)	-	-	-	0.0085	-
Muriate of potash (MOP)	-	-	-	-	0.0087


### Preparation of 30 mg kg^-1^ Arsenic Concentrate Soil

Collected soil samples were ground uniformly for sowing of mung bean seeds in pots. This background soil was measured at a 0.078 mg kg^-1^ concentration of As. The concentration of As in background soils (0.078 mg kg^-1^) was increased to 30 mg kg^-1^ through the addition of sodium arsenite (AsNaO_2_) as a source of As. A 0.0518 g sodium arsenite (AsNaO_2_) was used for each kg of soil in the pots to get the required concentration of As (30 mg kg^-1^).

### Arbuscular Mycorrhizal Fungi (AMF)

Arbuscular mycorrhizal fungi samples were collected from the Department of Environmental Science at BSMRAU. These AMF samples were found mixed with soils and roots of the host plant of Sorghum and housed in brick made pits under the research field of Environmental Science at BSMRAU. A mixture of AMF in soil and roots was cultured in a concrete structured seed bed for multiplication as a source of AMF. Based on the total biomass (soil) in the pots, 4% AMF content soils were used for the reduction of As stress in mung bean crops. Mycorrhizas spores in the soil and root samples were observed by following the Wet Sieving and Decanting Method (Gerdemann and Nicolson, 1963).

### Nutrient Augmentation in Soils by Fertilizers for Growing Mung Bean Crops

Four kilograms of ground soils with 200 g Trichoderma-enriched biofertilizers were mixed together in each pot. According to the recommendations of the BARI, Urea 50 kg ha^-1^, TSP 85 kg ha^-1^, and MOP 35 kg ha^-1^ were incorporated into the soil in each pot. The percentages of total nitrogen (0.0115), phosphorus (0.0085), and potassium (0.0087) were added in each experimental pot with the synthetic fertilizers. Then, 7–10 mung bean seeds of each genotype were sowed in each pot during the third week of April in 2017.

### Treatments and Replications

Six genotypes of BARI-released mung beans were collected for this pot experiment. First, the three genotypes of BARI mung 3, BARI mung 5, and BARI mung 8 were selected based on their dissimilar height. With these genotypes there were six treatments followed [T_1_ = rice husk BC, T_2_ = cow dung BC, T_3_ = sawdust BC, T_4_ = AMF mixed, T_5_ = selenium (15 mg kg^-1^), and T_6_ = control]. All soil was prepared at an As concentration of 30 mg kg^-1^. Five replications were used in this pot experiment and the total number of pots was 90. Then, another three genotypes of BARI mung 4, BARI mung 6, and BARI mung 7 were taken for the next stage of the experiment. In this stage, two treatments of T_1_ = AMF, T_2_ = control were used and five replications followed. All soil was also at an As concentration of 30 mg kg^-1^. A total number of 30 pots were used. All the treated materials were mixed with the soil based on 4% of the total biomass in each pot. As a final point, these six mung bean genotypes were grown in 0.078 mg kg^-1^ As concentrate background soils in pot. Five replications also followed in this stage for a total number of 30 pots.

### Sample Collection for the Determination of Stress Enzymes at Week 7

Mung bean leaves were collected during week 7 for the analysis of biochemical parameters. Leaves were collected from each treated pot separately and kept in Ziploc bags with proper labeling. During the collection of the leaves, all samples were kept in ice boxes and then brought into the laboratory for the sample extractions and the analysis of the biochemical parameters. Before extraction of leaf samples, all samples were preserved in a -20°C refrigerator.

### Analysis of Chlorophyll Content

Chlorophyll contents of mung bean leaves were determined following Arnon’s (1949) method. In this method, a 100 mg fresh leaf sample was homogenized in 80% acetone (80 ml acetone + 20 ml distilled water to make 100 ml) followed by centrifugation at 3,000 r/min for 5 min; the volume was then 5 ml with 80% acetone. Absorbance was recorded at 645 and 663 nm with a spectrophotometer (model-723N Japan). Chlorophyll a, b and total chlorophyll contents were calculated using the following formula:

$$\text{Chlorophyll a }(mg/gm/frwt)=(0.0127\times A_{663})-(0.00269\times A_{645}).$$

$$\text{Chlorophyll b }(mg/gm/frwt)=(0.0229\times A_{645})-(0.00468\times A_{663}).$$

$$\text{Total chlorophylls }(mg/gm/frwt)=(0.0202\times A_{645})+(0.00802\times A_{663}).$$

### Analysis of Proline Content

Proline content was measured according to Bates et al. (1973). An aliquot of fresh leaf (0.5 g) was homogenized in 10 ml of 3% sulfosalicylic acid, and the homogenate was centrifuged at 5,000 r/min for 15 min. A total of 2 ml supernatant was incubated with 2 ml acid ninhydrin (1.25 g ninhydrin dissolved in 30 ml glacial acetic acid and 20 ml of 6 mol L^-1^ phosphoric acid) and 2 ml glacial acetic acid for 1 h at 100°C, and then quickly cooled in an ice bath. The colored reaction mixture was extracted with 4 ml toluene, and the absorbance was recorded at 520 nm. The proline content was expressed using the following formula as the fresh weight basis, μmol g^-1^ fresh weight = (μg ml^-1^ proline × volume of toluene × volume of sulfosalicylic acid)/(0.5 × 115.5), where 115.5 is the molecular weight of proline and 0.5 is the sample weight.

### Analysis of Catalase Activity

Catalase content in mung bean leaves was estimated by the method of Aebi (1984). The assay mixture contained 0.750 ml of 50 mM potassium phosphate buffer (pH 7) and 0.1 ml enzyme extract. A reaction was initiated by adding 0.1 ml of 100 mM H_2_O_2_. Disappearance of H_2_O_2_ was monitored by measuring a decrease in absorbance at 240 nm for 2 min. Activity was calculated by using an extinction coefficient of 40 M^-1^ cm^-1^.

### Statistical Analysis

The design of this experiment followed the Completely Randomized Design (CRD). ANOVA and mean comparison of treatment effects on antioxidant activities under As stress were analyzed using R software.

## Results

### Chemical Properties of Background Samples

Total As concentrations in cow dung BC, Trichoderma-enriched biofertilizer, background soils, sawdust BC, and rice husk BC were 0.044, 0.04464, 0.0784, 0.00349, and 0.02499 mg kg^-1^, respectively. The percentages of organic carbon were 15.88, 14.50, 0.39, 24.60, and 6.32 in cow dung BC, Trichoderma-enriched biofertilizer, background soils, sawdust BC, and rice husk BC, respectively. Meanwhile, cow dung BC, Trichoderma-enriched biofertilizer, background soils, sawdust BC, rice husk BC, and urea fertilizer supplied 1.19, 1.28, 0.05, 0.33, 0.30, and 0.0115% of nitrogen, respectively. Likewise, cow dung BC, Trichoderma-enriched biofertilizer, background soils, sawdust BC, rice husk BC, and TSP fertilizers each supplied 1.28, 1.20, 0.001, 0.00, 0.10, and 0.0085% of phosphorus, respectively. Finally, the percentages of potassium added from cow dung BC, Trichoderma-enriched biofertilizer, background soils, sawdust BC, rice husk BC, and muriate of potash fertilizers were 1.42, 0.87, 0.003, 0.77, 0.33, and 0.0087, respectively.

### Chlorophyll

According to ANOVA, the treatment effect on chlorophyll a, b and total chlorophyll was found to be significantly different in 30 mg kg^-1^ As-concentrated soils in BARI mung 3, 4, 5, 6, 7, and 8. Interactions of mung bean varieties and treatments were also found to be significantly different with chlorophyll a, b, and total chlorophyll content (Tables 2, 4). The T_2_ treatment effect on chlorophyll a in BARI mung 3 crops was found to be significantly different from T_1_ and control. This T_2_ treatment effect on chlorophyll a was found to be statistically similar to T_3_-, T_4_-, and T_5_-treated BARI mung 3 crops. However, the T_4_ treatment effect on chlorophyll a in BARI mung 5 and 8 both was found significantly different (*p* ≤ 0.05) from their control. Similarly, the T_4_ treatment effect on chlorophyll b and total chlorophyll content was found to be significantly higher than control in BARI mung 5 crops *(p* ≤ 0.05) (Table 3). Chlorophyll a and b both were found to be significantly higher (*p* ≤ 0.05) in T_1_-treated BARI mung 4, 6, and 7 genotypes (Table 5). In background soils, chlorophyll content was found to be higher in BARI mung 7 and BARI mung 8 genotypes compared to other genotypes (Table 6).

**Table 2:** ANOVA on changes of antioxidants defense mechanism during the mitigation of arsenic uptake in BARI mung 3, BARI mung 5, and BARI mung 8 genotypes in soils with an arsenic concentration of 30 mg kg^-1^.

	Chlorophyll a				Chlorophyll b				Total chlorophyll			
			
	df	Sum of squares (SS)	Mean sum of square (MSS)	Pr(>F)	df	SS	MSS	Pr(>F)	df	SS	MSS	Pr(>F)
Variety	2	0.0001	7.5195e-05	1.692e-11 ^∗∗∗^	2	2.8032e-05	1.4016e-05	2.241e-12 ^∗∗∗^	2	0.0012	0.00064	6.743e-15 ^∗∗∗^
Treatment	5	0.0001	2.0400e-05	4.205e-07 ^∗∗∗^	5	1.2837e-05	2.5674e-06	1.405e-05 ^∗∗∗^	5	0.0007	0.00015	5.035e-09 ^∗∗∗^
Variety : Treatment	10	0.00014	1.4529e-05	1.837e-07 ^∗∗∗^	10	1.5173e-05	1.5173e-06	9.958e-05 ^∗∗∗^	10	0.0014	0.00014	9.248e-12 ^∗∗∗^
Residuals	72	0.00015	2.1060e-06		72	2.5330e-05	3.5180e-07	2.241e-12 ^∗∗∗^	72	0.0008	0.00001	6.743e-15 ^∗∗∗^

	**Proline (µg/g FW)**						**Catalase (mM/min/g FW)**					
		
	df	Sum of squares (SS)		Mean sum of square (MSS)		Pr(>F)	df	Sum of squares (SS)		Mean sum of square (MSS)		Pr(>F)

	2	0.05934		0.029668		0.0009573 ^∗∗∗^	2	0.00313		0.00157		0.76493
	5	0.66783		0.133566		<2.2e-16 ^∗∗∗^	5	2.00651		0.40130		<2e-16 ^∗∗∗^
	10	0.15531		0.015531		0.0002171 ^∗∗∗^	10	0.10215		0.01021		0.08513^α^
	72	0.27858		0.003869			72	0.41932		0.00582		


*^∗∗∗^ indicates significant difference at 0.1% (*p* ≤ 0.001) level of significance. ^α^ indicates significant difference at 10% (*p* ≤ 0.1) level of significance.*

**Table 3:** Mean comparison of treatment effects on antioxidant defense activities during the mitigation of arsenic uptake in BARI mung 3, BARI mung 5, and BARI mung 8 genotypes in soils with an arsenic concentration of 30 mg kg^-1^.

Variety and treatment	Chlorophyll a	Chlorophyll b	Total chlorophyll	Proline (μg/g FW)	Catalase (mM/min/g FW)
BARI mung 3: Rice husk biochar (T_1_)	0.0039 ± 2.382100e-04 a	0.0018 ± 5.683309e-05 a	0.0050 ± 0.000372 ab	0.3328 ± 0.03478 bc	0.3818 ± 0.02068 ab
BARI mung 3: Cow dung biochar (T_2_)	0.0081 ± 3.165502e-04 bcde	0.0037 ± 3.157467e-04 bcdef	0.0198 ± 0.003405 efg	0.2928 ± 0.02083 ab	0.3296 ± 0.04771 ab
BARI mung 3: Sawdust biochar (T_3_)	0.006 ± 6.046487e-05 abcd	0.0029 ± 3.533327e-04 abc	0.0080 ± 0.00067 abc	0.286 ± 0.00119 ab	0.3694 ± 0.0215 ab
BARI mung 3: AMF (T_4_)	0.006 ± 1.137981e-04 abcd	0.0036 ± 1.206400e-04 bcdef	0.00856 ± 0.0003 abc	0.3015 ± 0.0212 abc	0.690 ± 0.02001 d
BARI mung 3: Selenium -15 mg/kg (T_5_)	0.0051 ± 3.390192e-04 ab	0.0032 ± 2.207623e-04 abcde	0.0072 ± 0.00074 abc	0.3377 ± 0.00780 bc	0.6288 ± 0.06761 cd
BARI mung 3: T_6_ (control)	0.004 ± 1.187603e-04 a	0.0025 ± 4.297325e-04 ab	0.0022 ± 0.00100 a	0.5593 ± 0.02451 d	0.295 ± 0.01807 a
BARI mung 5: Rice husk biochar (T_1_)	0.009 ± 1.854023e-04 de	0.0041 ± 3.720215e-04 cdefg	0.016 ± 0.00196 defg	0.2572 ± 0.02125 ab	0.334 ± 0.03112 ab

BARI mung 5: Cow dung biochar (T_2_)	0.008 ± 3.046079e-04 bcde	0.0040 ± 3.709771e-04 cdefg	0.0127 ± 0.0002 bcde	0.18198 ± 0.02982 a	0.319 ± 0.02370 ab
BARI mung 5: Sawdust biochar (T_3_)	0.0086 ± 1.818553e-04 cde	0.0040 ± 4.069398e-05 cdefg	0.0210 ± 0.00374 fg	0.30432 ± 0.0509 abc	0.331 ± 0.03645 ab
BARI mung 5: AMF (T_4_)	0.0112 ± 5.683168e-04 e	0.0053 ± 1.607047e-04 g	0.0235 ± 0.0008 g	0.3262 ± 0.03348 bc	0.625 ± 0.03796 cd
BARI mung 5: Selenium -15 mg/kg (T_5_)	0.008 ± 7.348469e-06 cde	0.004 ± 3.154616e-04 efg	0.020 ± 0.00098 fg	0.2661 ± 0.01897 ab	0.699 ± 0.0396 d
BARI mung 5: T_6_ (control)	0.0062 ± 8.741875e-04 abc	0.0039 ± 1.619074e-04 cdef	0.0115 ± 0.0004 bcd	0.5184 ± 0.01630 d	0.319 ± 0.02383 ab

BARI mung 8: Rice husk biochar (T_1_)	0.0106 ± 1.799333e-04 e	0.0046 ± 3.061470e-04 fg	0.0131 ± 0.0007 cdef	0.1650 ± 0.00748 a	0.328 ± 0.02300 ab
BARI mung 8: Cow dung biochar (T_2_)	0.0055 ± 1.034118e-04 abc	0.0034 ± 1.872325e-04 bcdef	0.00797 ± 0.0004 abc	0.3489 ± 0.01964 bc	0.309 ± 0.00377 a
BARI mung 8: Sawdust biochar (T_3_)	0.0062 ± 2.679627e-04 abc	0.003 ± 3.021854e-04 bcdef	0.0081 ± 0.00054 abc	0.2820 ± 0.02383 ab	0.488 ± 0.0588 bc
BARI mung 8: AMF (T_4_)	0.0103 ± 2.373780e-03 e	0.0043 ± 3.193807e-04 cdef	0.01740 ± 0.0018 defg	0.26761 ± 0.0583 ab	0.620 ± 0.02365 cd
BARI mung 8: Selenium -15 mg/kg (T_5_)	0.0087 ± 3.793363e-04 cde	0.0037 ± 4.985980e-05 bcdef	0.0209 ± 0.00225 fg	0.2365 ± 0.02332 ab	0.658 ± 0.03290 cd
BARI mung 8: T_6_ (control)	0.0067 ± 3.731809e-04 abcd	0.0030 ± 1.922602e-04 abcd	0.0097 ± 0.00032 abcd	0.4432 ± 0.0229 cd	0.306 ± 0.02010 a


*Mean ± SE with different lower case letter(s) indicates significant difference at *p* ≤ 0.05.*

### Proline

According to the ANOVA, the treatment effect on proline was found to be significantly different (*p* ≤ 0.001) in soils with an As concentration of 30 mg kg^-1^ in BARI mung 3, 4, 5 6, 7, and 8 crops (Tables 2, 4). Proline contents were found to be significantly lower (*p* ≤ 0.05) than control in T_1_- to T_5_-treated BARI mung 3, BARI mung 5, and BARI mung 8 genotypes in soils with an As concentration of 30 mg kg^-1^ (Table 3). Additionally, T_2_-treated BARI mung 6 crops showed higher stress (*p* ≤ 0.05) due to high proline as compared to T_1_ treatment. This treatment effect on proline content was also found statistically similar with T_2_-treated BARI mung bean crops without AMF (Table 5). On the other hand, proline contents in BARI mung 3, 4, 7, and 8 crops were found statistically similar in background As-concentrated soils (Table 6).

**Table 4:** ANOVA on the effect of AMF on antioxidant defense mechanism in BARI mung 4, BARI mung 6, and BARI mung 7 genotypes in soils with an arsenic concentration of 30 mg kg^-1^.

	Chlorophyll a				Chlorophyll b				Total chlorophyll			
			
	df	Sum of squares (SS)	Mean sum of square (MSS)	Pr(>F)	df	SS	MSS	Pr(>F)	df	SS	MSS	Pr(>F)
Variety	2	4.6640e-06	2.3320e-06	0.004578 ^∗∗^	2	1.0597e-05	5.2983e-06	4.927e-08 ^∗∗∗^	2	2.8740e-04	1.437e-04	6.716e-07 ^∗∗∗^
Treatment	1	4.7278e-05	4.7278e-05	1.965e-11 ^∗∗∗^	1	8.1850e-06	8.1850e-06	8.926e-08 ^∗∗∗^	1	1.7750e-05	1.775e-05	0.0791^α^
Variety : Treatment	2	2.9660e-06	1.4830e-06	0.024906 ^∗^	2	3.4020e-07	1.7010e-07	0.3244	2	1.7100e-07	8.500e-08	0.9840
Residuals	24	8.2330e-06	3.4300e-07	0.004578 ^∗∗^	24	3.4584e-06	1.4410e-07		24	1.2667e-04	5.278e-06	6.716e-07 ^∗∗∗^

	**Proline (µg/g FW)**						**Catalase (mM/min/g FW)**					
		
	df	Sum of squares (SS)		Mean sum of square (MSS)		Pr(>F)	df	Sum of squares (SS)		Mean sum of square (MSS)		Pr(>F)

	2	0.0480		0.024024		0.0018734 ^∗∗^	2	0.027		0.0139		0.02904 ^∗^
	1	0.0412		0.041218		0.0009577 ^∗∗∗^	1	0.264		0.2646		5.308e-09 ^∗∗∗^
	2	0.0229		0.011450		0.0333136 ^∗^	2	0.003		0.0017		0.60410
	24	0.0698		0.002911			24	0.081		0.0033		0.02904 ^∗^


*^∗∗∗^ indicates significant difference at 0.1% (*p* ≤ 0.001) level of significance, ^∗∗^ indicates significant difference at 1% (*p* ≤ 0.01) level of significance, ^∗^ indicates significant difference at 5% (*p* ≤ 0.05) level of significance, ^α^ indicates significant difference at 10% (*p* ≤ 0.1) level of significance.*

**Table 5:** Mean comparison of AMF effects on antioxidant defense activities in BARI mung 4, BARI mung 6, and BARI mung 7 genotypes in soils with an arsenic concentration of 30 mg kg^-1^.

Variety and treatment	Chlorophyll a	Chlorophyll b	Total chlorophyll	Proline (μg/g FW)	Catalase (mM/min/g FW)
BARI mung 4: AMF (T_1_)	0.0051 ± 0.00039 bc	0.004 ± 3.084153e-04 c	0.0064 ± 0.0003 a	0.199 ± 0.033 ab	0.369 ± 0.0425 c
BARI mung 4: non-AMF (T_2_)	0.003 ± 0.00000 a	0.0036 ± 1.840761e-04 b	0.00470 ± 0.00141 a	0.238 ± 0.0258 abc	0.157 ± 0.0131 a
BARI mung 6: AMF (T_1_)	0.0048 ± 0.00021 b	0.003 ± 6.303967e-05 b	0.0071 ± 0.000275 a	0.164 ± 0.01128 a	0.424 ± 0.0251 c
BARI mung 6: non-AMF (T_2_)	0.0024 ± 0.00002 a	0.002 ± 9.159694e-05 a	0.00554 ± 0.00027 a	0.3166 ± 0.0175 c	0.232 ± 0.0250 ab
BARI mung 7: AMF (T_1_)	0.0062 ± 0.00026 c	0.0036 ± 1.019804e-04 b	0.01314 ± 0.00201 b	0.2970 ± 0.0317 bc	0.343 ± 0.0071 bc
BARI mung 7: non-AMF (T_2_)	0.0029 ± 0.00037 a	0.0024 ± 1.454441e-04 a	0.01180 ± 0.00020 b	0.3283 ± 0.0152 c	0.183 ± 0.0279 a


*Mean ± SE with different lower case letter(s) indicates significant difference at *p* ≤ 0.05.*

**Table 6:** Mean comparison of antioxidants activities at back ground soils (0.078 mg kg^-1^ arsenic) of different mung bean genotypes.

Varieties	Chlorophyll a	Chlorophyll b	Total chlorophyll	Proline (μg/g FW)	Catalase (mM/min/g FW)
BARI mung 3	0.0067 ± 8.366600e-04 b	0.003 ± 0.0003 a	0.0117 ± 0.0015 bc	0.1921 ± 0.04024 bc	0.419 ± 0.10271 abc
BARI mung 4	0.0038 ± 6.109010e-04 d	0.003 ± 0.00047 b	0.0067 ± 0.00102 d	0.1932 ± 0.01923 bc	0.484 ± 0.12976 a
BARI mung 5	0.0057 ± 7.684074e-04 c	0.003 ± 0.00016 b	0.008 ± 0.000701 cd	0.2471 ± 0.0334 a	0.354 ± 0.0787 bc
BARI mung 6	0.0042 ± 6.886799e-04 d	0.0032 ± 0.0006 b	0.0068 ± 0.00216 d	0.16675 ± 0.0273 c	0.306 ± 0.0509 c
BARI mung 7	0.0081 ± 3.114482e-05 a	0.0043 ± 0.00010 a	0.016 ± 0.00384 a	0.20691 ± 0.03085 b	0.416 ± 0.1323 abc
BARI mung 8	0.0063 ± 4.453426e-04 bc	0.004 ± 0.00074 a	0.0148 ± 0.00327 ab	0.21706 ± 0.0182 ab	0.476 ± 0.0539 ab


*Mean ± SE with different lower case letter(s) indicates significant difference at *p* ≤ 0.05.*

### Catalase

According to the ANOVA, the treatment effect on CAT was found to be significantly different (*p* ≤ 0.001) in soils with an As concentration of 30 mg kg^-1^ in BARI mung 3, 4, 5, 6, 7, and 8 crops (

## Discussion

Arsenic triggers the production of ROS, which have detrimental impacts on plants at biochemical, physiological, and molecular levels. The role of different enzymatic (SOD, CAT, glutathione reductase, and ascorbate peroxidase) and non-enzymatic [salicylic acid (SA), proline, phytochelatins, glutathione, nitric oxide, and phosphorous] substances under As stress have been defined via conceptual models showing As translocation and toxicity pathways in plants (Abbas et al., 2018). Several biochemical compounds such as chlorophyll, CAT, and proline were found to be significantly changed in mung bean crops under As stress (Swarnakar, 2014). The changes of these compounds create negative impacts on the growth and development of mung bean crops as well as other food crops. Among these parameters, increases in chlorophyll enhance the photosynthesis in addition to increasing the green color in food crops. BC, AMF, and Se work against As uptake in mung bean crops and contribute to a remarkable improvement in chlorophyll content (Tables 3, 5). However, As stress in mung bean crops was decreased due to the application of these As relief substances, and photosynthetic pigments increased in all treated mung bean genotypes. These pigments are potentially increased by the release of N and P from BC (Fiaz et al., 2014). Similarly, an addition of BC reduced heavy metal uptake and improved shoot/root growth, dry biomass, and enhanced the chlorophyll and carotenoid concentrations in food crops. The incorporation of BC improved the soil health, for example by accelerating other enzymatic activities under heavy metal stress conditions (Fiaz et al., 2014; Ali et al., 2017). The enzymatic activities of the leaves were enhanced after the application of BC, which could alleviate the oxidative stress posed by As (Table 3). Similar to previous studies in food crops, the chlorophyll contents were also enhanced after the addition of BC in mung bean crops (Kazemi et al., 2010). Addition of BC to soils enhances the crops’ yield due to the improvement of chlorophyll content. Therefore, BC could be used to improve the soil, displace conventional fossil fuel-based fertilizers, and sequester carbon, including increasing the green color of leaves with a higher volume of chlorophyll content (David and John, 2013).

Biochars such as rice husk, cow dung and sawdust have a comparable effect on the CAT enzyme in mung bean genotypes under As stress (Table 3). Similarly, research was conducted on the effect of BC and found a significant (*p* ≤ 0.05) effect on the CAT activity in food crops. In addition, BCs have significant potential for the reduction of heavy metal uptake in food crops, as well as in mung bean crops under As stress conditions (Yang et al., 2016). Research was also conducted on the effect of BC in wheat crops and higher CAT activities were found, in comparison to the control (Zhangliu et al., 2014). In contrast, CAT activity was found to be roughly similar under As stress in mung bean crops as compared with As-free background soils during the application of BC (Tables 3, 6). As a result, CAT activity was found higher in BC-applied soils due to its connectivity with ROS rather than the control in this study. Thus, BC reduced oxidative stress in As contaminated plants (Farhangi-Abriz and Torabian, 2017).

On the other hand, proline could enhance oxidative damages and osmolytes in As-stressed mung bean crops (Swarnakar, 2014). BC, AMF, and Se were applied for the reduction of osmotic and oxidative stress under As stress in mung bean crops. In this study, proline, an indicator of osmotic stress, was found to be significantly different in BC applied soils under As stress conditions in comparison with the control in mung bean crops (Table 3). This BC works against As stress as an inhibitor of As. Due to this effect, proline content was reduced in mung bean crops. For instance, BC reduces proline content in saline soil in wheat crops (Sidra et al., 2018). Similarly, proline content in mung bean crops in As stress conditions was reduced by the application of BC. Thus, BC is an effective treatment for the reduction of proline content as well as sugar in food crops. This means BC works against environmental stresses such as As, drought and salinity in the food crops (Yousara et al., 2017). Similar results were also found through the application of BCs such as rice husk and cow dung, which enhanced crop growth in addition to decreasing the stress of As in mung bean crops in this study.

The mycorrhizal relationship improves nutrient uptake and nutrient balance in plants and soils, respectively (Shi et al., 2017). AMF enhance the productivity of sweet basil plants under environmental stress conditions as well as As stress in mung bean plants (Salwa et al., 2016). The mycorrhizal inoculation significantly increased chlorophyll content and water use efficiency under stress conditions (Khalid et al., 2017). Moreover, AMF conferred tolerance to low temperatures in cucumber plants (Chen et al., 2013). In this study, AMF alleviated phytotoxicity and influenced the chlorophyll content under As stress conditions in mung bean crops (Table 3). The content of chlorophyll a, chlorophyll b, and carotenoid increased in *Panicum turgidum* plants along with mung bean crops due to the inoculation of AMF (Hashem et al., 2015). Thus, AMF have significant effects on the mitigation of As in these crops and could also increase crop productivity. Chlorophyll is one of the most important productivity indicators for food crops and was enhanced by the application of AMF in soils.

In addition to chlorophyll, mycorrhiza has a significant effect on CAT in the mung bean crops under As stress conditions (Table 3). Additionally, other plants such as *Gmelina arborea* species were influenced through AMF, increasing CAT under environmental stress. This AM fungus (*G. fasciculatum*) is highly effective against heavy metals and other environmental stresses in food crops as well as mung bean crops (Mayura et al., 2011). Defense mechanisms are enhanced through CAT for the protection of the plants from oxidative stress. ROS scavenging is one of the common defense responses against abiotic stresses in crops. ROS decreased due to the application of mycorrhiza in soils of mung bean crops (Vranova et al., 2002). As a mitigation to the As stress in mung bean crops, mycorrhiza is potentially helpful for alleviating oxidative stress as well as decreasing the toxic substances (Prochazkova et al., 2001; Shrivali et al., 2003). This activity highly influences the motivation of CAT activities in this study. Recently, Das and Sarkar (2018) also conducted similar research on chlorophyll and CAT activities in mung bean crops using bacterial strain *Acinetobacter lwoffii* (RJB-2) as well as AMF under As stress. Chlorophyll content and CAT activities were both found to be higher in microbial-treated mung bean crops alongside increased biomass accumulation and reduced oxidative damages.

Proline is an important biochemical index relating to stress response in food crops. The application of beneficial fungi reduced As stress, which in turn decreased the stress marker proline in mung bean leaves. For example, proline content was significantly reduced due to the application of mycorrhiza in citrus plants as well as in mung bean crops (Ying-Ning et al., 2013). These results also suggest that osmotic stress is also significantly reduced in AMF-applied mung bean crops (Swarnakar, 2014). Similar research was conducted on Alfalfa crops, and the researchers found that mycorrhiza reduced proline content alongside amelioration of As toxicity (Kanwal et al., 2015). Davies et al. (2000) observed the effect of mycorrhizae on pepper crops and determined the effect of the AMF (*Glomus intraradices*) and phosphorus availability on the accumulation of organic solutes, plant growth, cell membrane stability, and mineral nutrient acquisition in pepper plants at different levels of stress. In this instance, the content of proline also decreased due to the application of AMF, while the other antioxidant activity increased, supporting our current observation in mung bean crops in As-free conditions of background soils (Tables 3, 5, 6).

Similarly, Se also works against environmental stress in food crops. Arsenic is one of the important stressors in the production of mung bean crops in Bangladesh as well as in many regions of the world. For increasing the productivity of mung bean crops, it is important to block the uptake of As metal. Due to the mitigation approach by Se, plant growth increased through increasing chlorophyll content in mung bean crops (Cartes et al., 2010). Arsenic is a heavy metalloid that causes severe adverse effects on the growth and development of plants, while Se is another metalloid and a beneficial element when present in appropriate amounts. It was observed that the presence of Se along with As increased seed germination, root–shoot growth, total chlorophyll and protein contents compared with only As stress (Chandana et al., 2015). Similarly, chlorophyll content was found significantly higher in As stress conditions because of the use of Se for blocking As translocation toward the shoot.

Selenium enhances tolerance to stress in a range of plant species (Yin et al., 2019). Arsenic stress was reduced by the application of Se in mung bean crops. As a result, ROS levels were deferred by the application of Se, which was highly connected with CAT activity (Emma et al., 2015). Due to the reduction of toxicity using Se, CAT activity was found higher in Se-applied soils than the control in mung bean crops under As stress conditions (Table 2). This Se activity was found to be significantly positive against As stress, including reductions in ROS as well as CAT activity (Kumar et al., 2014). On the other hand, proline content was found to be significantly lower in Se-treated mung bean crops than the control in As stress conditions (Table 3). Proline is an indicator of any abiotic stress—including As stress—in food crops (Shamsul et al., 2012). A positive correlation was found between proline accumulation and plant stress. Proline, an amino acid, plays a highly beneficial role in plants exposed to various stress conditions as well as As stress. It was found that proline content significantly differed in some genotypes of mung bean crops as compared to the control through the application of Se in As stress conditions. Thus, soil amendment with Se can be an effective approach for the reduction of proline content as well as osmolytes in mung bean crops under As stress conditions.

The productivity of food crops depends not only on the prevalence of abiotic stress but also on the ecological components. Several ecological factors reduce the growth, development and biomass production in food crops. In this aspect, endophytic fungi (AMF) can play a vital role in the reduction of ecologically negative impacts as well as in the improvement of the nutritional quality through the reduction of ROS in food crops. It can be suggested that endophytic fungi and their pragmatic approach might be important for sustainable food and agriculture (Rai et al., 2014). The detoxification of heavy metals through endophytic fungi also reduces MDA and H_2_O_2_ content in food crops and increases CAT, peroxidase, and chlorophyll content (Mallick et al., 2014). SA and Se both are significantly effective in increasing the activities of CAT and SOD under heavy metal stress in food crops (Song and Yang, 2014; Alyemeni et al., 2017). It is concluded that AMF, BC, and Se are all significantly effective for the reduction of physiological damage to food crops under As stress in soils.

## Conclusion

Chlorophyll, CAT, and proline are some of the most important biochemical compounds in food crops for their roles in plant growth, development, and responses to environmental stresses. Generally, abiotic stresses affect plant growth and development and As pollution is a common environmental stress in the South Asia region for the cultivation of food crops. Despite being an important pulse crop throughout the world, mung bean production is largely threatened by As stress in Bangladesh and other regions in the sub-continent. In this regard, BC, AMF, and Se were used for the mitigation of As uptake as well as to study the enzymatic activities during As stress conditions. The tested substances were able to increase the chlorophyll content in mung bean crops. CAT activity was found to be increased in comparison with the control due to application of BC, AMF, and Se in mung bean crops. This indicates that the oxidative stress was drastically reduced in As-stressed crops due to the application of As relief substances. Moreover, proline was reduced in AMF-applied soils rather than the control under As stress conditions. AMF is highly effective against As stress in comparison to BC and Se. It is concluded that BC, AMF and Se are all highly effective against As stress and can be used to reduce As-induced oxidative stress in mung bean.

## Data Availability

All datasets generated for this study are included in the manuscript and/or the supplementary files.

## Author Contributions

MA led as a principal researcher for the requirement of his Ph.D. dissertation and he was responsible for the conduction of research and writing. MdH was responsible for supervising this research jointly. RM provided constructive suggestion on the development of research plan. LC-B provided constructive guidelines for the improvement of design, statistical analysis, syntax formation, and coherence of this manuscript. GA contributed to the improvement of this manuscript through proofreading as a subject specialist.

## Conflict of Interest Statement

The authors declare that the research was conducted in the absence of any commercial or financial relationships that could be construed as a potential conflict of interest.
